# Ultrafast one‐pass FASTQ data preprocessing, quality control, and deduplication using fastp

**DOI:** 10.1002/imt2.107

**Published:** 2023-05-08

**Authors:** Shifu Chen

**Affiliations:** ^1^ HaploX Biotechnology Shenzhen China; ^2^ Shenzhen Institutes of Advanced Technology Chinese Academy of Sciences Shenzhen China

**Keywords:** adapter, duplication, FASTQ, filtering, preprocessing, quality control

## Abstract

A large amount of sequencing data is generated and processed every day with the continuous evolution of sequencing technology and the expansion of sequencing applications. One consequence of such sequencing data explosion is the increasing cost and complexity of data processing. The preprocessing of FASTQ data, which means removing adapter contamination, filtering low‐quality reads, and correcting wrongly represented bases, is an indispensable but resource intensive part of sequencing data analysis. Therefore, although a lot of software applications have been developed to solve this problem, bioinformatics scientists and engineers are still pursuing faster, simpler, and more energy‐efficient software. Several years ago, the author developed fastp, which is an ultrafast all‐in‐one FASTQ data preprocessor with many modern features. This software has been approved by many bioinformatics users and has been continuously maintained and updated. Since the first publication on fastp, it has been greatly improved, making it even faster and more powerful. For instance, the duplication evaluation module has been improved, and a new deduplication module has been added. This study aimed to introduce the new features of fastp and demonstrate how it was designed and implemented.

## Highlights

- Fastp is an ultrafast tool that processes FASTQ data in a single pass.

- Fastp has been redesigned to make it faster and generate reproducible results.

- Fastp introduces efficient FASTQ‐level deduplication without sorting the reads.

## Graphical abstract

Fastp is a widely adopted tool for FASTQ data preprocessing and quality control. It is ultrafast and versatile and can perform adapter removal, global or quality trimming, read filtering, unique molecular identifier processing, base correction, and many other actions within a single pass of data scanning. Fastp has been reconstructed and upgraded with some new features. Compared to fastp 0.20.0, the new fastp 0.23.2 is even 80% faster.

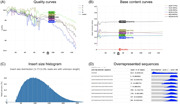

## INTRODUCTION

High‐throughput sequencing technology has developed rapidly for nearly 20 years. Every year, various new sequencing platforms are launched, and the sequencing throughput continues to increase. Regardless of the sequencing platform and the sequencing throughput, FASTQ is adopted as the standard for the generated data of most high‐throughput sequencing platforms. These FASTQ data need to go through quality control and a series of preprocessing steps before they can enter the downstream analysis to ensure the cleanness and accuracy of the data. In almost all application scenarios of sequencing, the effectiveness of data preprocessing usually greatly impacts the final analysis results [1]. For example, circulating tumor DNA sequencing can be used for finding personalized therapy and detecting minimal residual disease. However, its result is seriously affected by sequencing data quality, as adapter contamination, sequencing noises, and other artifacts can impact the analysis accuracy, leading to incorrect treatment decisions [2].

**Figure 1:**
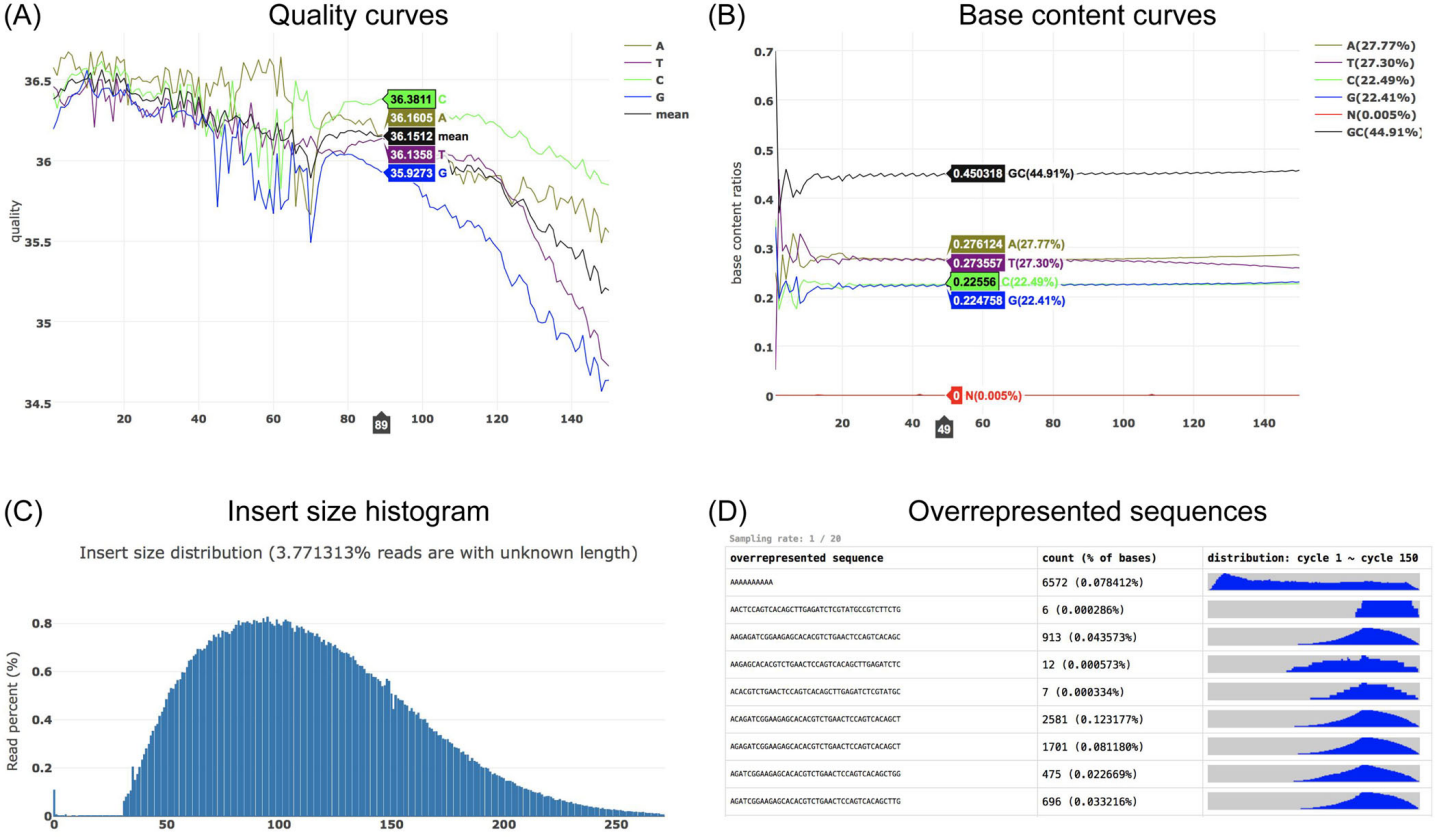
Part of interactive statistical plots of fastp. (A) The per‐cycle quality curves, and (B) the per‐cycle base content curves. (C) The distribution of evaluated insert size, with a small portion of reads remaining unknown due to their paired reads that are not overlapped, which is usually due to the fragments being too long. (D) The statistics of overrepresented sequences, including their per‐cycle distribution.

Many tools have been developed to address the problem of FASTQ data preprocessing and quality control. For example, Cutadapt [3] and Trimmomatic [4] have been widely used for adapter trimming and quality pruning. Many tools, such as FQC Dashboard [5] and NGS QC Toolkit [6], were developed for FASTQ data quality control. However, these tools have two major problems. One is that they are not efficient enough or consume too much memory. The other is that their features are not comprehensive enough, resulting in the need to go through the data multiple times with different software modules to complete the whole preprocessing and QC process. Fastp [7], which is an ultrafast all‐in‐one FASTQ data preprocessor with many modern features, was developed to solve these problems. Fastp can perform adapter removal, global or quality trimming, read filtering, unique molecular identifier processing, base correction, and many other actions within a single pass of data scanning. Since the first publication of fastp, it has been adopted by many community users. However, the earlier versions of fastp have some problems; for example, the execution results cannot be reproduced, the data compression speed is not ideal, and so on. The new fastp was developed by reconstructing the multithreaded computing architecture of fastp software and introducing a more efficient data compression and decompression algorithm, which is based on the highly optimized compression library igzip, to solve the aforementioned key problems. Besides architecture optimization, some new features have also been added to the new fastp, such as rapid deduplication. Fastp outputs an interactive HyperText Markup Language (HTML) report for manual checking and an informative JSON report for automatic quality control. Figure 1 shows a part of fastp's HTML report.

## METHODS

**Figure 2:**
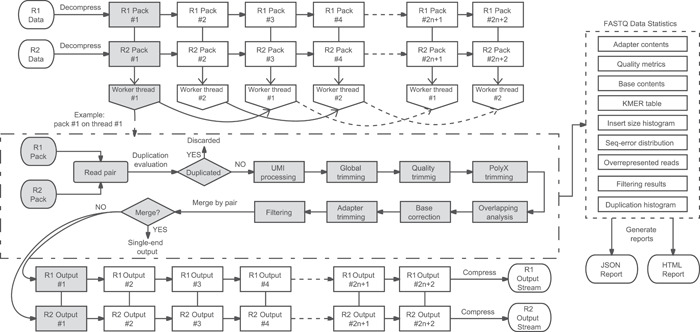
Paired‐end data processing workflow of fastp. The workflow can be simply divided into a decompressor, a preprocessor, and a compressor. The input‐paired FASTQ files are decompressed individually to read packs, and each pack consists of fixed read records. Each worker thread picks the odd or even read packs one by one, processes the reads, makes some statistics, and outputs the clean data to the compressor in the same order.

Fastp is a multithreaded multifunctional preprocessor for FASTQ streams. It accepts single‐end or paired‐end FASTQ data as inputs and outputs the processed data along with the QC metric reports. Figure 2 shows how fastp processes paired‐end FASTQ data.

Two worker threads are used for demonstration, but usually, much more worker threads (usually 3–16) are used to make preprocessing faster. The classical producer/consumer thread model is applied, and specifically, the input and output read packs are stored in a single‐producer‐single‐consumer (SPSC) list for thread‐safe communications. This SPSC list is implemented without any thread locks to support high‐performance interthread communication. As shown in Figure 2, for a certain read pack, it is fixed that in which worker thread the read pack will be processed. This feature keeps the output and input data in the same order, making the output completely reproducible, which means the resulting output files will be identical if the command is run twice.

Most features shown in Figure 2 were introduced in the first publication on fastp. Some features, such as paired‐end merging and deduplication, have been recently introduced. Applying paired‐end merging is relatively simple after the overlapping analysis is complete.

**Figure 3:**
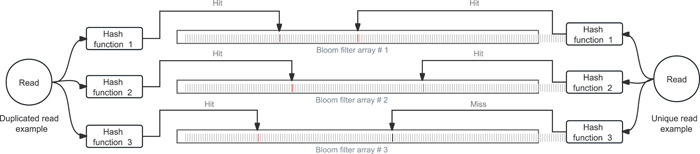
How fastp determines whether a read is unique or duplicated.

Removing redundant reads is a necessary step for the NGS data analysis pipeline. Previous deduplication tools typically require the reads to be mapped to the reference genome first, which makes them inefficient and unsuitable for some applications that do not invoke sequence alignment. The new fastp implements a fast, accurate, and memory‐efficient FASTQ‐level deduplication. Figure 3 briefly illustrates the method by which fastp removes duplicated reads.

As shown in Figure 3, many bloom filter arrays (e.g., three) are used, and each has *L* bits. A hash function is defined accordingly for each array. A hash function maps a read sequence into an integer number *p* ∈ [0, *L*); therefore, a read *R* will be mapped to *p*_1_, *p*_2_, and *p*_3_. If Array1[*p*_1_], Array2[*p*_2_], and Array3[*p*_3_] are all positive, then *R* is marked as duplicated; otherwise, Array1[*p*_1_], Array2[*p*_2_], and Array3[*p*_3_] are set to be positive. For paired‐end reads, the read pairs are combined first and then treated as same as single‐end reads.

## RESULTS AND DISCUSSIONS

Compared with earlier versions, the new fastp provided more powerful performance and generated reproducible results. Although some new features have been added and speed has been improved, fastp still maintains a very small memory requirement. Typically, it requires only 4GB or less memory to run fastp, which makes it very suitable for cloud‐based applications. Table 1 shows the performance comparison between Trimmomatic‐0.39, fastp 0.20.0, and fastp 0.23.2.

**Table 1:** Comparative performance of Trimmomatic‐0.39, fastp 0.20.0, and fastp 0.23.2.

ID	Platform	File size (GB)	Bases (billion)	Trimmomatic‐0.39	Fastp 0.20.0	Fastp 0.23.2
S1	MGI	33.670	99.577	3:51:02	0:42:38	0:25:15
		34.079				
S2	Illumina	6.926	29.380	0:56:23	0:12:06	0:06:01
		7.111				
S3	MGI	15.659	40.448	1:39:50	0:19:10	0:10:50
		13.373				
S4	MGI	6.512	16.401	0:41:13	0:08:00	0:04:26
		5.447				
S5	Illumina	4.160	17.625	0:33:43	0:11:12	0:03:44
		4.336				
S6	Illumina	1.310	5.557	0:10:17	0:02:04	0:01:09
		1.348				
S7	MGI	2.929	7.909	0:19:57	0:03:51	0:02:09
		2.752				
S8	MGI	1.595	4.090	0:10:09	0:01:49	0:01:07
		1.360				
S9	MGI	20.200	57.615	2:21:14	0:31:39	0:15:14
		18.727				
S10	MGI	0.861	3.677	0:06:43	0:01:21	0:00:44
		0.891				
S11	Illumina	9.359	41.674	1:13:16	0:17:41	0:07:37
		9.462				

A total of 11 paired‐end sequencing data, which were generated from Illumina or MGI platforms, were evaluated on a typical computing server (CPU, 64‐cores 2.5 GHz; RAM, 1024 GB). All experiments were conducted using eight worker threads with the default or recommended command options. As shown in Table 1, fastp 0.23.2 was ~9× faster than Trimmomatic‐0.39, and ~1.8× faster than fastp 0.20.0. This result indicated that the new fastp took only 25 min to perform preprocessing and QC of paired‐end data of 100 billion bases, which was usually the amount of whole‐genome sequencing data.

## CONCLUSION

The new architecture significantly enhanced the performance of fastp, making the results of fastp reproducible. In addition, fastp was extremely easy to get started with and could be easily obtained by downloading the prebuilt binaries or installing it via BioConda [8]. Considering its ultrahigh performance, rich functions, and simple usage, fastp should be one of the best choices for FASTQ data preprocessing and quality control.

## AUTHOR CONTRIBUTIONS

Shifu Chen developed the software and wrote the manuscript.

## CONFLICT OF INTEREST STATEMENT

The author declares no conflict of interest.

## Data Availability

No data are available. The fastp software is a part of the OpenGene project. Its source code and prebuilt binaries can be found at https://github.com/OpenGene/fastp. Supplementary materials (figures, tables, scripts, graphical abstract, slides, videos, Chinese translated version, and update materials) may be found in the online DOI or iMeta Science https://www.imeta.science/.
